# Intergenerational relational dynamics in co-parenting families: a cross-sectional analysis through a systemic theoretical lens

**DOI:** 10.3389/fpsyg.2026.1748675

**Published:** 2026-04-22

**Authors:** Ziran Li, Mohd Najmi Daud, Mohamad Salleh Bin Abdul Ghani, Asmidawati Ashari

**Affiliations:** Department of Human Development and Family Studies, Faculty of Human Ecology, Universiti Putra Malaysia, Serdang, Selangor, Malaysia

**Keywords:** intergenerational co-parenting, grandparent–parent relationships, family environment, systemic theory, family cohesion, co-parenting conflict, child behavioral outcomes, Chinese families

## Abstract

**Introduction:**

Intergenerational co-parenting, where parents and grandparents share child-rearing responsibilities, is prevalent in Chinese families. Despite its cultural significance, few studies have examined how grandparent parent co-parenting relationship dimensions relate to broader family functioning using a systemic theoretical framework.

**Methods:**

A cross-sectional survey was conducted with 710 Chinese parents (303 fathers, 407 mothers), of whom 426 reported active grandparent involvement in child-rearing. The Co-Parenting Relationship Scale (CRS) and Family Environment Scale (FES) were administered. Data were analyzed using hierarchical multiple regression and bootstrap mediation analysis (PROCESS macro, Model 4, 5,000 resamples).

**Results:**

Negative co-parenting dimensions (particularly undermining, M = 4.61) scored higher than positive dimensions. Co-parenting support significantly predicted overall family environment quality (*β* = 0.243, *p* < 0.05). Co-parenting debasement negatively predicted family cohesion (*β* = –0.275, *p* < 0.01), while division of labor positively predicted cohesion (*β* = 0.224, *p* < 0.05). Bootstrap mediation analysis revealed that family environment partially mediated the relationship between co-parenting conflict and child behavioral difficulties (indirect effect = 0.038, 95% CI [0.011, 0.074])

**Discussion:**

Findings support systemic theoretical propositions regarding relational interdependence, structural boundaries, and circular causality within co-parenting families. Co-parenting relationship quality is significantly associated with family environment, which serves as a pathway influencing child outcomes. Limitations include the cross-sectional design and reliance on self-report measures.

## 1 Introduction

### 1.1 Research background

The increasing diversification of family structures has contributed to the growing prevalence of co-parenting arrangements in which caregiving responsibilities are shared among parents, grandparents, and other extended family members. While such multigenerational caregiving can provide substantial support in child-rearing, it also introduces complex intergenerational dynamics that may give rise to conflict (Li et al., 2023; Xiao and Loke, 2021, 2022). These conflicts—rooted in divergent values, parenting beliefs, disciplinary philosophies, and power dynamics—can undermine family cohesion and adversely affect children’s psychological well-being (Li et al., 2020; Yang et al., 2022; Camisasca et al., 2022).

Intergenerational conflict within co-parenting families extends beyond the parent–child dyad to encompass tensions between grandparents and parents, and sometimes among other extended family members. Such conflicts can impede communication, erode mutual understanding, and diminish emotional cohesion, thereby threatening the stability and functioning of the family as a whole (Jung and Kim, 2016; Kim et al., 2010; Ling et al., 2023). In co-parenting contexts—where multiple caregivers collaboratively raise children across generational lines—maintaining healthy relational dynamics requires open dialogue, mutual respect for differing perspectives, and a collective commitment to prioritizing children’s developmental needs (Li and Wei, 2018).

Against this backdrop, systemic theory offers a valuable analytical framework for understanding complex familial dynamics (Ramchandani and Iles, 2014). Rooted in general systems theory and cybernetics, the systemic perspective conceptualizes the family as an interconnected whole, emphasizing relational patterns, feedback loops, and the structural organization of family subsystems (Nichols and Tafuri, 2013; Dietz et al., 2015). A critical distinction must be drawn here: Systemic Family Therapy (SFT) refers to a clinical modality involving therapist-facilitated sessions and structured techniques aimed at restructuring maladaptive relational patterns (Carr, 2019). The present study does not implement or evaluate SFT. Rather, it draws upon systemic theoretical principles—particularly the constructs of relational interdependence, structural boundaries, hierarchical organization, and circular causality—as an interpretive lens through which naturally occurring family dynamics in co-parenting households are examined (Barbato et al., 2020; Cunningham et al., 2023; Gabb and Singh, 2015).

This study is therefore exploratory in nature, aiming to examine associations between grandparent-parent co-parenting relational factors and family environment indicators through a systemic theoretical lens. Consistent with this orientation, the following research questions guide the inquiry:

*RQ1: How are grandparent-parent co-parenting relational factors associated with family environment indicators in co-parenting families?*
*RQ2: To what extent are the observed associative patterns consistent with core systemic theoretical principles of family functioning?*

### 1.2 Research objectives and significance

The primary objective of this study is to investigate associative relationships between grandparent-parent co-parenting relational dynamics and family environment characteristics, interpreted through a systemic theoretical framework. The study focuses on measurable relational constructs including co-parenting support, conflict, undermining behaviors, and their associations with family cohesion and communication patterns.

Multigenerational co-parenting is particularly prevalent in Chinese cultural contexts, where intergenerational caregiving remains a deeply embedded social norm (Xie et al., 2022; Zhang et al., 2020). While this structure offers the potential for rich familial support, it also presents significant relational challenges. Intergenerational conflicts typically arise from disparities in educational philosophies, value systems, parenting beliefs, and communication styles (Li et al., 2020; Yang et al., 2022). Understanding these dynamics through a systemic theoretical lens may yield insights that are not readily captured by individual-level or dyadic analytical approaches.

Academically, this study contributes to the theoretical understanding of intergenerational processes in co-parenting households by examining family relational dynamics at the systems level and providing an empirically grounded analysis of how systemic constructs manifest in naturally occurring family interactions (Carr, 2019; Wittenborn and Holtrop, 2022). Practically, the exploratory associations identified may inform family practitioners’ conceptualization of multigenerational family processes, although direct clinical recommendations require further empirical validation (Andolfi, 2024).

## 2 Theoretical framework

### 2.1 Systemic theory as an analytical framework

Systemic theory, originating from general systems theory and cybernetics, provides a framework for understanding the family as a complex, dynamic, and interdependent structure comprising multiple interconnected subsystems (Chiang and Bai, 2022; Ulbricht et al., 2013). The family system typically encompasses marital, parent–child, and grandparent-grandchild subsystems, each performing distinct functions. Disruptions or changes in one subsystem can trigger cascading effects across the entire system, altering overall family equilibrium (Dallos and Vetere, 2014).

Three core systemic constructs are particularly relevant to the present study. First, relational interdependence posits that family members’ behaviors, emotions, and cognitions are mutually influential, such that individual functioning cannot be fully understood in isolation from the broader relational context (Berry et al., 2019). Second, structural organization refers to the hierarchical arrangement of family subsystems, including boundaries between subsystems, role distributions, and power dynamics that govern family interactions (Andolfi, 2024). Third, circular causality suggests that family interactions operate through reciprocal feedback loops, whereby relational behaviors are both causes and consequences of systemic patterns, rather than following simple linear causal chains (Carr, 2019).

SFT encompasses specific clinical modalities—including structural, Milan systemic, and experiential approaches such as family sculpting (Satir, 1972; Cigoli and Scabini, 2006; Selvini Palazzoli et al., 1978; Boscolo et al., 1987)—that are facilitated by trained clinicians in therapeutic settings. The present study employs systemic theoretical constructs as interpretive lenses through which self-reported family relational data are analyzed, rather than evaluating any clinical modality.

### 2.2 Intergenerational co-parenting: conceptual background

Co-parenting refers to the collaborative sharing of caregiving responsibilities among multiple adults in a family (Cabrera et al., 2012). In the specific context of intergenerational co-parenting, grandparents and parents jointly participate in daily caregiving, educational guidance, and the transmission of cultural norms across generations (Han et al., 2020; Xu et al., 2023b). This arrangement, particularly prevalent in Chinese families, can strengthen intergenerational bonds while simultaneously introducing relational tensions stemming from divergent parenting approaches, value systems, and role expectations (Han et al., 2020).

The intergenerational co-parenting framework encompasses five core dimensions: power and authority, division of labor, conflict, coping and adaptation, and reciprocity (Xu et al., 2023a; Zhu et al., 2024). These dimensions reflect the relational complexities inherent in grandparent-parent caregiving arrangements and differentiate intergenerational co-parenting from other family models. Changes in one dimension can disrupt caregiving responsibilities and provoke conflicts over role clarity, necessitating adaptive strategies to maintain family stability (Xiao and Loke, 2021).

While intergenerational tensions can produce emotional distress and communication breakdowns, they may also function as catalysts for relational growth and role renegotiation when resolved constructively (Mak et al., 2021). Effective conflict resolution encourages family members to articulate their values and emotional needs, fostering empathy and mutual understanding, and enhancing the family’s long-term adaptive capacity (Attar-Schwartz, 2015; Yang et al., 2021).

### 2.3 Operationalization: linking systemic constructs to empirical indicators

A key methodological requirement in applying systemic theory to empirical research is establishing explicit operational links between theoretical constructs and measured variables. Table 1 presents the mapping between the systemic theoretical constructs guiding this study and the specific empirical indicators used in the survey instruments.

**Table 1:** Operationalization of systemic theoretical constructs.

Systemic construct	Operational definition	Empirical indicator
Relational interdependence	Degree to which co-parenting behaviors mutually influence family functioning	CRS: Support, Intimacy, Approval
Structural boundaries	Clarity and permeability of role boundaries between generational subsystems	CRS: Division of labor, Agreement; FES: Organization, Control
Hierarchical organization	Distribution of authority and decision-making power across generational lines	CRS: Undermining, Conflict; FES: Control
Circular causality	Reciprocal feedback patterns linking co-parenting quality and family environment	CRS: Conflict, Debasement; FES: Cohesion, Conflict, Emotional Expression

CRS = Co-Parenting Relationship Scale; FES = Family Environment Scale.

This operationalization table makes transparent the theoretical reasoning underlying the selection of survey instruments and subscales, and provides readers with a clear framework for interpreting the empirical results in relation to systemic theory.

## 3 Method

### 3.1 Research design and participants

This study employed a cross-sectional survey design. Data were collected via an online self-report questionnaire distributed through multiple digital platforms, targeting families engaged in co-parenting arrangements.

A total of 850 responses were received, of which 140 were excluded due to incomplete or demonstrably careless responding (e.g., uniform response patterns across all items), yielding a final sample of 710 valid respondents (303 fathers, 407 mothers). Among these, 426 respondents reported that their children were raised with active grandparent involvement in caregiving, defined as grandparents regularly participating in daily childcare activities within multigenerational households. Children in these households spanned early to middle childhood (approximately 2–12 years; M ≈ 7 years).

In terms of socioeconomic characteristics, the majority of respondents reported having completed tertiary education (bachelor’s degree or above: 58.7%; junior college: 24.2%; high school or below: 17.1%). Regarding household income, 46.8% of families reported a monthly household income above ¥10,000 RMB, 35.5% between ¥5,000 and ¥10,000, and 17.7% below ¥5,000. Most participants resided in urban areas (87.3%), with the remainder from suburban or semi-urban regions. These demographic characteristics are broadly consistent with the profile of Chinese urban families engaged in multigenerational co-parenting arrangements (Zhang et al., 2020), although the online convenience sampling method may overrepresent higher-educated and urban populations.

The sample was community-based and non-clinical; participants were not recruited from clinical settings nor experiencing clinically significant distress. This sampling approach is consistent with the study’s aim to examine everyday co-parenting dynamics.

### 3.2 Analytic samples

To ensure transparency, two analytic samples are distinguished throughout:

Full sample (*N* = 710): All valid respondents. Used for Family Environment Scale (FES) descriptive analyses, as all respondents completed this instrument regardless of caregiving arrangement.

Grandparent-involved subsample (*n* = 426): Respondents reporting active grandparent participation in caregiving. Only this subsample completed the Co-Parenting Relationship Scale (CRS). All regression and mediation analyses were conducted exclusively on this subsample. Each table explicitly identifies the analytic sample used.

### 3.3 Measures

#### 3.3.1 Co-parenting relationship scale (CRS)

The CRS consists of 35 items across eight dimensions: division of labor, co-parenting agreement, co-parenting intimacy, co-parenting conflict, co-parenting support, co-parenting approval, and co-parenting undermining, and co-parenting debasement. Items are rated on a 7-point Likert scale (1 = completely inconsistent to 7 = very consistent). The instrument was adapted and validated for grandparent-parent co-parenting dynamics in Chinese families (Li and Wei, 2018). Internal consistency for the overall scale was adequate (Cronbach’s *α* = 0.85), with subscale alphas ranging from 0.71 to 0.86.

#### 3.3.2 Family environment scale (FES)

The FES comprises 90 true/false items organized into 10 subscales assessing distinct dimensions of the family social environment: cohesion, emotional expression, conflict, independence, achievement orientation, intellectual-cultural orientation, recreational orientation, moral-religious emphasis, organization, and control (Moos and Moos, 1994). Higher scores indicate stronger manifestations of the corresponding characteristic.

Internal consistency for the FES subscales in the present sample ranged from *α* = 0.24 (independence) to α = 0.75 (cohesion). Table 2 presents the reliability coefficients.

**Table 2:** Internal consistency reliability of family environment scale subscales (*N* = 710).

Subscale	Cronbach’s *α*	Classification
Cohesion	0.75	Acceptable
Emotional expression	0.58	Marginal
Conflict	0.62	Marginal
Independence	0.24	Poor
Achievement orientation	0.41	Poor
Intellectual-cultural orientation	0.53	Marginal
Recreational orientation	0.47	Poor
Moral-religious emphasis	0.52	Marginal
Organization	0.56	Marginal
Control	0.44	Poor

Classifications follow conventional thresholds: acceptable (α ≥ 0.70), marginal (0.50 ≤ α < 0.70), poor (α < 0.50).

Several FES subscales displayed poor to marginal internal consistency, a known limitation of the FES true/false format, particularly in non-Western cultural contexts (Boyd et al., 2009; Sanford et al., 1999). Notably, the Chinese adaptation of the FES by Fei et al. (1991) reported a comparable range of subscale reliability (*α* = 0.32–0.76) in Chinese community samples, and Phillips et al. (1998) confirmed similar factor structures with variable subscale consistency in Chinese psychiatric research contexts, suggesting that lower reliability on certain subscales reflects cultural and item-format factors rather than solely sample-specific issues. It should be noted that, despite these analytic precautions, the retention of marginal-reliability subscales (α = 0.52–0.62) in the composite family environment score may still introduce measurement error, potentially leading to slight attenuation of parameter estimates. Readers should therefore interpret effect sizes involving these subscales as conservative approximations.

Three analytic decisions were made in response. First, subscales with poor reliability (α < 0.50: independence, achievement orientation, recreational orientation, control) were excluded from primary regression and mediation analyses. Second, regression analyses focused on composite family environment scores and on the cohesion subscale (α = 0.75), which demonstrated acceptable reliability and represents a central construct in systemic theory. Third, supplementary robustness analyses excluding all marginal-reliability subscales (α < 0.60) confirmed the stability of the primary results.

#### 3.3.3 Ethical procedures

All procedures were conducted in accordance with institutional ethical standards. Informed consent was obtained electronically prior to participation. Participants were assured of anonymity and could withdraw at any time.

### 3.4 Analytic strategy

Data were analyzed using SPSS 26.0 in three stages:

Stage 1: Descriptive statistics. Means, standard deviations, and score distributions for all CRS and FES subscales were computed. CRS analyses used the grandparent-involved subsample (*n* = 426); FES analyses used the full sample (*N* = 710).
Stage 2: Multiple regression analysis. Hierarchical multiple regression models were estimated on the grandparent-involved subsample (n = 426) to examine associations between CRS dimensions (entered simultaneously) and family environment indicators. Assumptions of linearity, normality of residuals, homoscedasticity, and absence of multicollinearity were assessed; variance inflation factors (VIF) for all predictors were below 3.0.
Stage 3: Mediation analysis. The potential mediating role of the family environment was examined using the Baron and Kenny (1986) causal steps approach as a framework for evaluating mediation conditions. To address the known limitations of the causal steps method in providing direct statistical tests of indirect effects, bias-corrected bootstrap confidence intervals were computed using the PROCESS macro (Model 4; Hayes, 2018) with 5,000 bootstrap resamples. An indirect effect was deemed statistically significant if the 95% bias-corrected bootstrap confidence interval did not include zero. All mediation analyses were conducted on the grandparent-involved subsample (n = 426). Given the cross-sectional design, mediation results are interpreted as tests of statistical indirect effects rather than as evidence of causal mediation pathways (Maxwell and Cole, 2007).

## 4 Results

### 4.1 Descriptive statistics: co-parenting relational quality

Descriptive statistics for the eight CRS dimensions are presented in Table 3.

**Table 3:** Descriptive statistics of grandparent-parent co-parenting relationship dimensions (*n* = 426).

Dimension	*N*	Min	Max	M	SD
Co-parenting intimacy	426	1	7	3.67	1.79
Co-parenting agreement	426	1	7	3.79	1.77
Co-parenting conflict	426	1	7	3.84	1.51
Co-parenting debasement	426	1	7	3.73	1.48
Co-parenting support	426	1	7	3.77	1.68
Co-parenting approval	426	1	7	3.67	1.61
Co-parenting undermining	426	1	7	4.61	1.86
Division of labor	426	1	7	3.74	1.67

*n* = 426 (grandparent-involved subsample). 7-point Likert scale (1 = completely inconsistent to 7 = very consistent).

Negative co-parenting dimensions yielded higher mean scores than positive dimensions. Co-parenting undermining exhibited the highest mean across all dimensions (M = 4.61, SD = 1.86), indicating that mutual undermining of parenting authority was a salient feature of these relationships. Viewed through a systemic lens, this suggests potential disruptions in hierarchical organization and boundary maintenance across generational subsystems (see Table 1).

### 4.2 Regression analyses

Three hierarchical multiple regression models were estimated on the grandparent-involved subsample (*n* = 426).

#### 4.2.1 Model 1: overall family environment

Co-parenting support was the only significant predictor (*β* = 0.243, *p* = 0.022), consistent with the systemic construct of relational interdependence (Table 1). The model explained 19% of the variance in overall family environment. While this R^2^ value is modest in absolute terms, it falls within the typical range for exploratory family research examining complex, multidetermined outcomes (Fincham and Beach, 2010). The remaining 81% of unexplained variance likely reflects the influence of numerous unmeasured factors—including cultural norms governing intergenerational relationships, household economic pressures, grandparents’ physical and mental health status, neighborhood and community resources, and individual personality characteristics—that collectively shape the family environment but were beyond the scope of the present study (Table 4).

**Table 4:** Multiple regression: co-parenting dimensions predicting overall family environment (*n* = 426).

Predictor	B	SE	β	t	*p*
Co-parenting support	0.025	0.012	0.243	2.30	0.022*
Co-parenting approval	0.019	0.010	0.152	1.20	0.220
Co-parenting undermining	−0.005	0.012	−0.036	−0.61	0.540
Division of labor	0.014	0.016	0.124	1.10	0.270
Co-parenting intimacy	0.002	0.014	−0.056	−0.58	0.560
Co-parenting agreement	0.010	0.012	0.003	0.02	0.990
Co-parenting conflict	−0.011	0.010	−0.086	−0.82	0.410
Co-parenting debasement	−0.003	0.013	−0.057	−0.57	0.580

R^2^ = 0.19, F (7, 418) = 13.4, *p* < 0.001. DV: composite FES score (excluding subscales with α < 0.50). *p < 0.05.

#### 4.2.2 Model 2: family cohesion

Division of labor was positively associated with family cohesion (*β* = 0.224, *p* = 0.010), consistent with the systemic construct of structural boundaries (Table 1). Co-parenting debasement was negatively associated with cohesion (*β* = −0.275, *p* = 0.010), suggesting that mutual debasement contributes to self-reinforcing cycles of relational discord—consistent with the principle of circular causality. The model explained 7% of the variance in family cohesion (R^2^ = 0.07). This modest explanatory power indicates that co-parenting relational quality represents one of many contributing factors to family cohesion; unmeasured variables such as marital relationship quality, parenting self-efficacy, grandparents’ health and autonomy, and broader socioeconomic conditions likely account for substantial additional variance (Table 5).

**Table 5:** Multiple regression: co-parenting dimensions predicting family cohesion (*n* = 426).

Predictor	B	SE	β	t	*p*
Co-parenting support	0.009	0.012	0.084	0.65	0.520
Co-parenting approval	−0.006	0.013	−0.041	−0.35	0.720
Co-parenting undermining	−0.015	0.012	−0.059	−1.20	0.250
Division of labor	0.024	0.015	0.224	1.70	0.010*
Co-parenting intimacy	0.002	0.014	0.016	0.13	0.900
Co-parenting agreement	−0.050	0.012	−0.002	−0.01	0.100
Co-parenting conflict	0.016	0.011	0.162	1.40	0.150
Co-parenting debasement	−0.030	0.013	−0.275	−2.60	0.010**

R^2^ = 0.07, F (7, 418) = 3.9, *p* < 0.001. DV: FES cohesion subscale (α = 0.75). *p < 0.05. **p < 0.01.

#### 4.2.3 Model 3: family environment success indicators

Co-parenting support again emerged as the sole significant predictor (*β* = 0.255, *p* = 0.040), reinforcing the pattern observed in Model 1. The model explained only 4% of the variance in family environment success indicators (R^2^ = 0.04). This low explanatory power underscores that co-parenting dynamics capture only a small fraction of the factors influencing family success outcomes. Given the multidetermined nature of family functioning—shaped by economic resources, social support networks, individual resilience, cultural expectations, and numerous other contextual factors—the small R^2^ values across all three models should be interpreted as indicating that the identified co-parenting predictors represent statistically significant but practically modest unique contributions to family environment variation (Table 6).

**Table 6:** Multiple regression: co-parenting dimensions predicting family environment success indicators (*n* = 426).

Predictor	B	SE	β	t	*p*
Co-parenting support	0.024	0.012	0.255	2.00	0.040*
Co-parenting approval	−0.008	0.010	−0.071	−0.56	0.580
Co-parenting undermining	−0.004	0.012	−0.029	−0.53	0.600
Division of labor	−0.007	0.008	−0.096	−0.77	0.450
Co-parenting intimacy	0.002	0.010	0.007	0.07	0.950
Co-parenting agreement	0.016	0.013	0.141	1.30	0.180
Co-parenting conflict	0.010	0.010	0.084	0.76	0.450
Co-parenting debasement	−0.013	0.014	−0.121	−1.10	0.280

R^2^ = 0.04, F (7, 418) = 3.2, *p* < 0.001. *p < 0.05.

### 4.3 Mediation analysis

Mediation analysis (*n* = 426) examined whether the family environment mediates the association between co-parenting relational quality and behavioral outcomes. Using the PROCESS macro (Model 4; Hayes, 2018) with 5,000 bootstrap resamples, a significant indirect pathway was identified: intergenerational co-parenting conflict was associated with increased behavioral difficulties through the mediating role of the family environment. Specifically, the indirect effect of co-parenting conflict on children’s behavioral difficulties via the family environment was statistically significant (indirect effect = 0.038, Boot SE = 0.016, 95% bias-corrected bootstrap CI [0.011, 0.074], excluding zero). The direct effect of co-parenting conflict on behavioral difficulties, controlling for the family environment, remained significant (B = 0.112, SE = 0.046, *p* = 0.016), suggesting partial mediation—that is, the family environment accounts for a meaningful portion of the association (approximately 25.3% of the total effect), while a substantial direct pathway persists. When co-parenting conflicts are elevated, the family environment is characterized by lower cohesion and greater interpersonal tension, which in turn is associated with maladaptive coping strategies (Lebow and Rekart, 2007). From the perspective of triangulation—a systemic concept describing how unresolved dyadic conflicts draw in third parties—unresolved grandparent-parent tensions may intensify children’s behavioral symptoms (Hughes and Asarnow, 2011; Klomek and Stanley, 2007).

The cross-sectional design precludes causal interpretation; the indirect effects should be understood as associations consistent with systemic theoretical propositions rather than as evidence of causal mechanisms (Maxwell and Cole, 2007).

## 5 Discussion

This study examined associations between grandparent-parent co-parenting relational factors and family environment indicators, drawing on systemic theory as an interpretive framework. Three principal findings emerged.

### 5.1 Co-parenting support and family environment quality

Across two regression models, co-parenting support emerged as a significant positive predictor of family environment quality (Model 1: *β* = 0.243, *p* < 0.05; Model 3: *β* = 0.255, *p* < 0.05). Interpreted through the systemic construct of relational interdependence (Table 1), this finding aligns with the proposition that supportive interactions within one subsystem are positively associated with broader family functioning (Andolfi, 2024; Carr, 2019). Supportive co-parenting dynamics may facilitate clearer communication, reduce role ambiguity, and promote shared purpose (Berry et al., 2019; Cepukiene and Neophytou, 2024).

More broadly, the modest R^2^ values observed across all three regression models (R^2^ = 0.19, 0.07, 0.04) warrant explicit interpretation. These values indicate that the seven co-parenting dimensions collectively explained only a small to moderate proportion of variance in family environment indicators. This pattern is not unexpected in family research, where outcome variables are shaped by a complex web of interacting factors operating at individual, relational, familial, and sociocultural levels (Cox and Paley, 2003). Specifically, unmeasured variables—including but not limited to cultural norms governing filial piety and grandparental authority, household economic stress, grandparents’ physical health and cognitive functioning, parental mental health, neighborhood safety, and the quality of broader social support networks—likely account for substantial portions of the unexplained variance. The present findings should therefore be understood as identifying specific, statistically reliable associations between co-parenting relational factors and family environment indicators, rather than as comprehensive models of family environment determination.

However, the cross-sectional design does not permit directional or causal conclusions. It is equally plausible that families with more favorable environments are better positioned to develop supportive co-parenting relationships, or that unmeasured third variables underlie both constructs simultaneously.

### 5.2 Co-parenting debasement, division of labor, and family cohesion

The negative association between co-parenting debasement and cohesion (*β* = −0.275, *p* < 0.01) is consistent with the systemic principle of circular causality, which posits that destructive relational behaviors create self-reinforcing cycles that progressively erode family functioning (Dallos and Vetere, 2014). Descriptive findings reinforce this: co-parenting undermining exhibited the highest mean score among all dimensions (M = 4.61), suggesting that mutual authority undermining is a salient feature of these relationships.

The positive association between division of labor and cohesion (*β* = 0.224, *p* < 0.05) aligns with the systemic construct of structural boundaries. Clearly delineated roles and boundaries between family subsystems may promote adaptive functioning by reducing role conflict and ambiguity (Nichols and Tafuri, 2013).

### 5.3 The family environment as a systemic pathway

Mediation analysis suggested that the family environment may serve as an intermediary through which co-parenting relational dynamics are associated with individual-level outcomes. The bootstrap-based indirect effect was statistically significant, with the 95% bias-corrected confidence interval excluding zero, providing quantitative evidence for this associative pathway beyond the traditional causal steps approach. This finding is theoretically consistent with systemic models that position the family environment as both a product of subsystem interactions and a context shaping individual adaptation (Chiang and Bai, 2022). However, cross-sectional mediation results must be interpreted with considerable caution, as Maxwell and Cole (2007) demonstrated that cross-sectional mediation estimates can be substantially biased relative to longitudinal estimates, and the temporal ordering of variables remains empirically unestablished.

### 5.4 Theoretical implications

The findings provide empirical support for the utility of systemic theory as an analytical framework for understanding intergenerational relational dynamics. The observed patterns are consistent with three core constructs: relational interdependence (support → environment quality), structural boundaries (division of labor → cohesion), and circular causality (co-parenting debasement → reduced cohesion). These findings demonstrate that systemic constructs can productively inform empirical family research at the observational level, independent of clinical application.

### 5.5 Practical implications

Family practitioners may find the identification of co-parenting support as a consistent positive predictor, and co-parenting debasement as a negative predictor of cohesion, relevant to case conceptualization when working with multigenerational families. These relational dynamics may represent meaningful targets for assessment and future research. Direct clinical recommendations, however, await validation through experimental research designs.

## 6 Limitations and future directions

Several limitations warrant acknowledgment:

First, the cross-sectional design precludes causal inferences. Future research should employ longitudinal designs with repeated measurements to establish temporal ordering and examine within-family change processes.

Second, exclusive reliance on self-report questionnaires introduces potential common-method variance and social desirability bias. Future studies would benefit from multi-informant designs and observational methods to triangulate findings.

Third, several FES subscales displayed poor to marginal internal consistency (*α* = 0.24 to 0.75). Although primary analyses were restricted to subscales with acceptable reliability (α ≥ 0.50) and robustness checks excluding all marginal-reliability subscales (α < 0.60) confirmed the stability of key findings, measurement limitations constrain the precision of parameter estimates. Specifically, the retention of marginal-reliability subscales (α = 0.52–0.62) in the composite family environment score may have introduced measurement error that attenuated observed effect sizes, meaning that true associations could be somewhat stronger than those reported. Future research should consider culturally adapted family environment measures with established cross-cultural psychometric properties, or employ latent variable modeling approaches that explicitly account for measurement error.

Fourth, the online convenience sampling strategy and restriction to the Chinese cultural context limit the generalizability of findings. The sample was recruited entirely through digital platforms, which may systematically overrepresent younger, more educated, and urban-dwelling parents. Although the sample’s socioeconomic profile (see Section 3.1) is broadly consistent with urban Chinese multigenerational families, it may not adequately represent rural families, lower-income households, or families from ethnic minority backgrounds—populations in which intergenerational co-parenting dynamics may operate differently. Cross-cultural replication studies, particularly in Western and other non-Chinese Asian contexts, are needed to evaluate the cultural specificity versus universality of the observed associations.

Fifth, no clinical intervention was implemented; the study examined naturally occurring family dynamics only. The theoretical compatibility of the observed associations with systemic principles should not be interpreted as evidence for clinical efficacy. Future experimental studies—employing randomized controlled designs and clinical outcome measures—are needed to bridge the gap between the relational patterns identified here and evidence-based practice.

Future research should prioritize: (a) longitudinal, multi-informant study designs; (b) culturally adapted and psychometrically robust measurement instruments; (c) mixed-method approaches combining quantitative surveys with qualitative interviews; and (d) experimental studies that directly test whether targeted family programs can modify the relational dynamics identified as significant predictors in the present findings.

## 7 Conclusion

This study examined associations between grandparent-parent co-parenting relational dynamics and family environment indicators in Chinese multigenerational families, employing systemic theory as an analytical framework. Three key findings emerged: (a) co-parenting support was consistently associated with higher family environment quality, consistent with relational interdependence; (b) co-parenting debasement was negatively associated with family cohesion, consistent with circular causality; and (c) division of labor was positively associated with family cohesion, consistent with structural boundaries.

These correlational findings provide empirical support for the utility of systemic theoretical constructs in analyzing intergenerational family dynamics, while transparently acknowledging the methodological constraints of a cross-sectional, self-report research design. This study contributes to the theoretical understanding of multigenerational co-parenting processes and provides a foundation for future longitudinal and experimental research aimed at advancing both scientific knowledge and evidence-based family practice.

## Data Availability

The raw data supporting the conclusions of this article will be made available by the authors, without undue reservation.
